# Clinical Significance of Various Drug-Sensitivity Markers in Patients with Surgically Resected Pulmonary Pleomorphic Carcinoma

**DOI:** 10.3390/cancers11111636

**Published:** 2019-10-24

**Authors:** Hisao Imai, Kimihiro Shimizu, Osamu Kawashima, Hideki Endoh, Kazuyoshi Imaizumi, Yasuhiro Goto, Mitsuhiro Kamiyoshihara, Masayuki Sugano, Ryohei Yamamoto, Shigebumi Tanaka, Atsushi Fujita, Yoshihito Kogure, Yukio Seki, Akira Mogi, Tetsunari Oyama, Koichi Minato, Takayuki Asao, Kyoichi Kaira

**Affiliations:** 1 Division of Respiratory Medicine, Gunma Prefectural Cancer Center, Gunma 373-8550, Japan; m06701014@gunma-u.ac.jp (H.I.); kminato@gunma-cc.jp (K.M.); 2 Departments of General Surgical Science, Gunma University Graduate School of Medicine, Gunma 371-8511, Japan; kmshimizu@gmail.com (K.S.); akmogi@gunma-u.ac.jp (A.M.); 3 Department of Respiratory Surgery, Shibukawa Medical Center, Gunma 377-0280, Japan; kawashima.osamu.ct@mail.hosp.go.jp; 4 Department of Thoracic Surgery, Saku Central Hospital Advanced Care Center, Nagano 385-0051, Japan; hidend0509@yahoo.co.jp (H.E.); yamamoto.ryohei@sakuhp.or.jp (R.Y.); 5 Department of Respiratory Medicine, Fujita Health University School of Medicine, Aichi 470-1192, Japan; jeanluc@fujita-hu.ac.jp (K.I.);; 6 Department of General Thoracic Surgery, Japanese Red Cross Maebashi Hospital, 371-0811, Japan; micha2005jp@yahoo.co.jp; 7 Department of Respiratory Surgery, Takasaki Medical Center, Gunma 370-0829, Japan; massugano@gmail.com; 8 Department of Respiratory Surgery, Isesaki Municipal Hospital, Gunma 372-0817, Japan; tanakasigebumi@yahoo.co.jp; 9 Division of Thoracic Surgery Gunma Prefectural Cancer Center, Gunma 373-8550, Japan; afujita@gunma-cc.jp; 10 Department of Respiratory Medicine, Nagoya Medical Center, Aichi 460-0001, Japan; yo-kogure@umin.ac.jp; 11 Department of Thoracic Surgery, Nagoya Medical Center, Aichi 460-0001, Japan; seki.yukio.kj@mail.hosp.go.jp; 12 Department of Diagnostic Pathology, Gunma University Graduate School of Medicine, Gunma 371-8511, Japan; oyama@gunma-u.ac.jp; 13 Big Data Center for Integrative Analysis, Gunma University Initiative for Advance Research, Gunma 371-8511, Japan; asao@gunma-u.ac.jp; 14 Department of Oncology Clinical Development, Gunma University Graduate School of Medicine, Gunma 371-8511, Japan; 15 Department of Respiratory Medicine, Comprehensive Cancer Center, International Medical Center, Saitama Medical University, Saitama 350-1298, Japan

**Keywords:** drug sensitivity, GRP78/BiP, stathmin 1, Topo-II, TS, TUBB3, VEGFR2, pleomorphic carcinoma, lung, immunohistochemistry

## Abstract

Various drug-sensitivity markers are potentially responsible for tumor progression and chemotherapy resistance in cancer patients with both epithelial and sarcomatous components; however, the clinicopathological significance of drug-sensitivity markers in patients with pulmonary pleomorphic carcinoma (PPC) remains unknown. Here, we clarified the prognostic impact of these drug-sensitivity markers in PPC by performing immunohistochemical and clinicopathologic analyses of samples from 105 patients with surgically resected PPC in order to evaluate levels of vascular endothelial growth factor 2 (VEGFR2), stathmin 1 (STMN1), tubulin β3 class III (TUBB3), thymidylate synthetase (TS), topoisomerase II (Topo-II), glucose-regulated protein, and 78 kDa (GRP78)/binding immunoglobulin protein (BiP). We observed the rates of high expression for VEGFR2, STMN1, TUBB3, TS, Topo-II, and GRP78/BiP were 33% (39/105), 35% (37/105), 61% (64/105), 51% (53/105), 31% (33/105), and 51% (53/105) of the samples, respectively. Moreover, multivariate analysis identified VEGFR2 and GRP78/BiP as significant independent markers for predicting worse prognosis. These findings suggested elevated VEGFR2 and decreased GRP78/BiP levels as independent factors for predicting poor outcomes following surgical resection in patients with PPC.

## 1. Introduction

Pulmonary pleomorphic carcinoma (PPC) is a rare epithelial tumor with aggressive characteristics and an incidence ranging from 0.1% to 0.4% of all lung cancers [1]. PPC contains carcinomatous and sarcomatoid components and is classified as a subtype of sarcomatoid carcinoma of the lung according to the World Health Organization histologic classification of lung neoplasms [2,3]. Previous studies report that PPC exhibits a more aggressive malignant potential than any other non-small cell lung cancer (NSCLC) and is closely linked to a worse prognosis and poorer response to systemic chemotherapy [4,5,6]. Because of its rarity, little is known about useful biomarkers for predicting treatment outcomes in patients with PPC. Systemic chemotherapy is indicated for patients with recurrent or advanced PPC; however, these patients frequently develop chemotherapeutic resistance. Moreover, relationships between this resistance and possible predictive markers remain unknown. Therefore, the selection of PPC patients exhibiting sensitivity to chemotherapy in order to allow the identification of predictive markers is necessary. 

Previous studies reported that levels of vascular endothelial growth factor receptor 2 (VEGFR2), stathmin 1 (STMN1), tubulin β3 class III (TUBB3), thymidylate synthase (TS), topoisomerase II (Topo-II), glucose-regulated protein, and 78 kDa (GRP78)/binding immunoglobulin protein (BiP) are closely associated with chemotherapeutic sensitivity in patients with various cancers. VEGFR2 is the main pro-angiogenic receptor for VEGF-A and plays a key role in tumor-induced angiogenesis [7], with another study demonstrating VEGFR2 expression in various tumor cells [8]. STMN1 is a cytosolic phosphoprotein that plays a crucial role in controlling cellular division and proliferation by regulating microtubule dynamics [9], as well as a variety of biological processes associated with carcinogenesis, with elevated STMN1 levels associated with cancer proliferation, resistance to taxanes, such as paclitaxel, and a poorer prognosis in a variety of human cancers [10]. Additionally, STMN1 is a possible marker of cancer cell proliferation. The isotype composition of β-tubulins is related to taxane-based chemotherapy responsiveness [11], and a recent review described a correlation between TUBB3 level and response to anti-microtubule agents in advanced cases of NSCLC [12]. Moreover, several clinical studies report that NSCLC patients with elevated TUBB3 levels exhibit greater resistance to paclitaxel/vinorelbine-based chemotherapy regimens than those with low TUBB3 levels [13]. Thymidylate synthase (TS) is an enzyme that plays an important role in DNA synthesis and catalyzes the methylation of deoxyuridine monophosphate to deoxythymidine monophosphate [14]. Additionally, TS is a target of 5-fluorouracil (5-FU), an anticancer chemotherapeutic agent used in the treatment of various human cancers [15] and closely associated with intratumoral expression of TS [16], which is significantly correlated with proliferative activity and poor prognosis in patients with NSCLC [17]. Topo-II, a target of amrubicin, represents a potential biomarker in various malignancies, including breast [18] and lung cancers [19,20]. A large-scale prospective pooled analysis reported Topo-II level as a significant biomarker for predicting the benefits of adjuvant anthracycline chemotherapy in breast cancer [18]. Furthermore, GRP78, also referred to as BiP, is a major molecular chaperone in the endoplasmic reticulum (ER) and present on the cell membrane and in the cytoplasm [21]. GRP78/BiP is involved in the folding and assembly of newly synthesized proteins in the ER and promotes resistance to ER-stress-induced apoptosis [21,22,23]. Although GRP78/BiP levels are elevated in many cancer cells and human cancers and closely associated with malignancy, metastasis, and chemotherapeutic resistance [22,23], few studies have evaluated its prognostic significance in patients with breast, lung, gastric, hepatocellular, or prostate cancer [23]. These markers are potentially responsible for tumor progression and chemotherapeutic resistance in cancer patients with both epithelial and sarcomatous components; however, their clinicopathological significance remains unclear in patients with PPC.

Here, we conducted a clinicopathologic investigation of VEGFR2, STMN1, TUBB3, TS, Topo-II, and GRP78/BiP levels in patients with surgically resected PPC. Because of its rare incidence and the difficulty in its diagnosis using biopsy samples, tumor samples of surgically resected PPC were collected from multiple institutions.

## 2. Results

### 2.1. Patient Characteristics and Immunohistochemistry

The percentages based on differential expression of the target markers are listed in Table 1, and patient demographics based on different markers expression are listed in Table 2. The median patient age was 69 years (range: 35–88 years), with 79 men (75%) and 26 women (25%). Eighty-four patients (80%) were smokers and 34 (32%) were diagnosed with stage I disease, 37 (35%) with stage II, 27 (26%) with stage III, and seven (7%) with stage IV. All patients were diagnosed using resected primary tumors. Histologic analysis revealed that 29 patients with PPC harbored a combination of carcinomatous and sarcomatous components. In the remaining 76 primary tumors, carcinomatous components were identified in 48 patients with adenocarcinoma, 13 with squamous cell carcinoma, 8 with adenosquamous cell carcinoma, 2 with small cell carcinoma, and 5 with large cell carcinoma. Of the sarcomatous components, 69 patients exhibited spindle-cell type, 10 giant-cell type, and 26 both spindle- and giant-cell types. The day of surgery was considered the start day for measuring postoperative survival, and the median follow-up period was 476 days (range: 30–4519 days).

**Figure 1:**
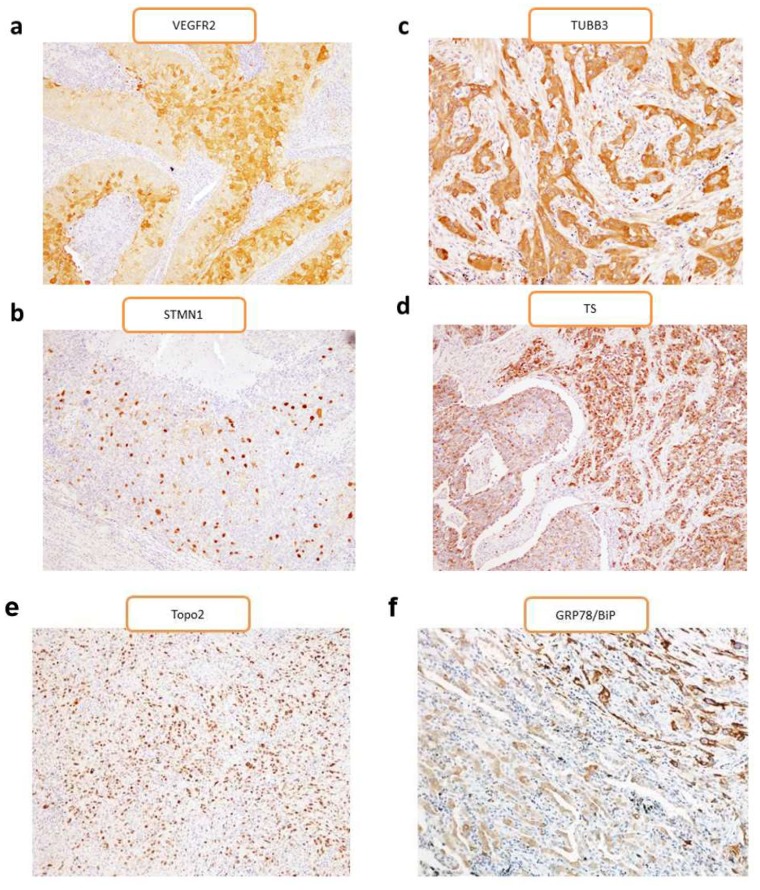
Representative image of immunohistochemical staining in patients with pulmonary pleomorphic carcinoma (PPC). (**a**) Immunopositivity for VEGFR2 in resected samples was observed mainly in the cytoplasm, with some samples also showing positive staining on the cellular membrane. (**b**) Immunopositivity for STMN1 showed a score of grade 4 and positive staining in the cytoplasm. (**c**) Immunopositivity for TUBB3 showed an H-score of 7 and positive staining in the cytoplasm. (**d**) Immunopositivity for TS showed a score of grade 5 and positive staining in nuclei and the cytoplasm. (**e**) Immunopositivity for Topo-II showed a score of grade 5 and positive staining in nuclei. (**f**) Immunopositivity for glucose-regulated protein and 78 kDa (GRP78/BiP) showed a score of grade 5 and positive staining in the cytoplasm. STMN1, stathmin 1; Topo-II, topoisomerase II; TS, thymidylate synthase; TUBB3, tubulin β3 class III; VEGFR2, vascular endothelial growth factor receptor 2. Original magnification, ×200.

We performed an immunohistochemical examination of the 105 primary samples from PPC patients, with representative findings for each of the markers presented in Figure 1. The percentages of high expression and average scores for VEGFR2, TUBB3, STMN1, Topo-II, TS, and GRP78/BiP are listed in Table 1. 

Our results showed the following: elevated VEGFR2 level was significantly linked with STMN1 level; elevated TUBB3 level was significantly associated with gender, vascular invasion, and TS; elevated STMN1 was significantly associated with VEGFR2 and TS levels; elevated Topo-II level was significantly associated with age and the T factor; elevated TS level was significantly associated with TUBB3, STMN1, and GRP78/BiP level; and elevated GRP78/BiP level was significantly associated with the N factor and pathological stage (Table 2).

### 2.2. Survival Analysis

**Figure 2:**
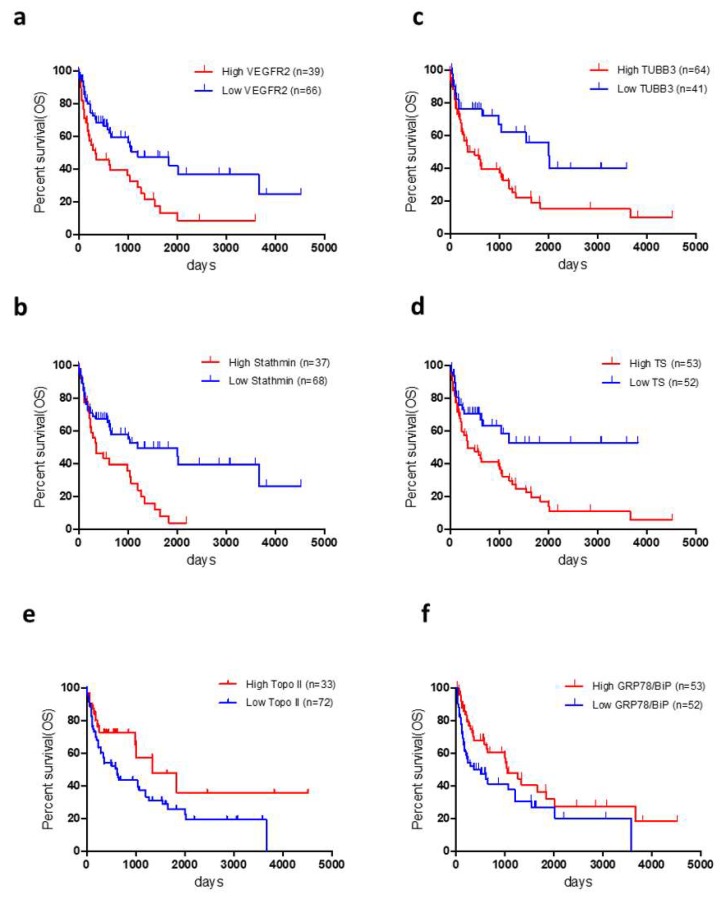
Kaplan-Meier survival curves for patients exhibiting differential marker levels. Kaplan-Meier plots showing OS according to (**a**) VEGFR2 level (VEGFR2-high/-low) [VEGFR2-high, median = 352 days; VEGFR2-low, median = 1209 days (log-rank: *p* < 0.01)], (**b**) STMN1 level (STMN1-high/-low) [STMN1-high, median = 359 days; STMN1-low, median = 1202 days (log-rank: *p* < 0.01)], (**c**) TUBB3 level (TUBB3-high/-low) [TUBB3-high, median = 361 days; TUBB3-low, median = 2010 days (log-rank: *p* < 0.01)], (**d**) TS level (TS-high/-low) [TS-high, median = 361 days; TS-low, median = not reached (log-rank: *p* < 0.01), (**e**) Topo-II level (Topo-II -high/-low) [Topo-II -high, median = 1338 days; Topo-II-low, median = 615 days (log-rank: *p* < 0.05)], and (**f**) GRP78/BiP level (GRP78/BiP-high/-low) [GRP78/BiP-high, median = 1047 days; GRP78/BiP-low, median = 507 days (log-rank: *p* = 0.04)]. OS, overall survival; STMN1, stathmin 1; Topo-II, topoisomerase II; TS, thymidylate synthase; TUBB3, tubulin β3 class III; VEGFR2, vascular endothelial growth factor receptor 2.

The median disease-free survival (DFS) and overall survival (OS) for all patients were 443 days and 991 days, respectively. The median DFS and OS for patients with adenocarcinoma and non-adenocarcinoma components were 522 days and 1038 days, and 336 days and 507 days, respectively. Of the 105 patients, 60 died after the initial surgery. Univariate and multivariate analyses were performed in all patients (Table 3; Table 4), with univariate analysis identifying disease stage and VEGFR2, TUBB3, STMN1, Topo-II, TS, and GRP78/BiP levels as significant prognostic markers for OS in all patients and disease stage, lymphatic permeation, pleural involvement, and VEGFR2 and STMN1 levels as significant predictors for DFS in all patients (Table 3). Application of a univariate log-rank test enabled screening of variables with a cut-off of *p* < 0.05, with subsequent multivariate analysis identifying disease stage and VEGFR2 and GRP78/BiP levels as independent prognostic factors for predicting a worse OS in all patients and disease stage, pleural involvement, and VEGFR2 and STMN1 levels as significant prognostic markers for DFS in all patients were (Table 3). Figure 2 shows the Kaplan–Meier survival curves for patients exhibiting high and low levels of VEGFR2, STMN1, TUBB3, TS, Topo-II, and GRP78/BiP, respectively.

We then performed univariate and multivariate analyses according to histological type. Univariate analysis identified disease stage and STMN1 and TS levels as significant prognostic markers for OS in patients with an adenocarcinoma component, and disease stage and VEGFR2 and TUBB3 levels as significant prognostic markers for OS in patients with a non-adenocarcinoma component. Additionally, significant predictors for DFS in patients with an adenocarcinoma component were STMN1 and GRP78/BiP levels, and significant predictors for DFS in patients with a non-adenocarcinoma component were disease stage and VEGFR2 level (Table 3). Furthermore, multivariate analysis confirmed disease stage as an independent prognostic factor for predicting worse OS in patients with an adenocarcinoma component and disease stage and VEGFR2 level as independent prognostic factors for predicting worse OS in patients with a non-adenocarcinoma component. Additionally, significant prognostic markers for DFS in patients with an adenocarcinoma component were STMN1 and GRP78/BiP levels and disease stage and VEGFR2 level in patients with a non-adenocarcinoma component (Table 4).

## 3. Discussion

To the best of our knowledge, this is the first study evaluating the clinicopathological significance of various drug-sensitivity markers, including VEGFR2, STMN1, TUBB3, TS, Topo-II, and GRP78/BiP, in patients with surgically resected PPC. We found that elevated VEGFR2 levels and decreased GRP78/BiP levels were independent prognostic variables for predicting a worse outcome after surgical resection. Additionally, the data suggested that VEGFR2 plays a crucial role in the survival of patients with non-adenocarcinoma-component PPC. PPC is recognized as a rare tumor with a dismal outcome, and there are few promising drug targets for its treatment; however, our findings suggest VEGFR2 and GRP78/BiP as potential target candidates.

In this study, VEGFR2 was expressed in 33% of all PPC patients, and the data suggested that elevated VEGFR2 level can be used as an independent prognostic variable for predicting a worse outcome after surgical resection. VEGFR2 is the main pro-angiogenic receptor for VEGF-A and plays a key role in tumor-induced angiogenesis [7]. These data suggest that VEGF-A and VEGFR2 play important roles in cancer-cell physiology. Interestingly, previous reports showed VEGFR2 expression in 30% to 91% of tumor cells [24,25] along with associated poor prognosis [24,26]. Another study showed that compared with other NSCLCs, 18F-fluorodeoxyglucose (18-FDG) uptake, glucose metabolism, angiogenesis, and cell proliferation were significantly elevated in patients with PPC [5], with 18F-FDG uptake significantly correlated with VEGF expression, which is an important prognostic indicator in various cancers [27]. Angiogenesis is essential for tumor growth, and enhanced vascular supplies of nutrients and oxygen are reflective of malignant potential. Our results suggested that VEGFR2 levels might offer prognostic value to aid in the identification of patients as potential responders to antiangiogenic therapies, such as bevacizumab and ramucirumab.

We found that GRP78/BiP was expressed in 51% of the PPC patients, and that a low level of GRP78/BiP represented an independent prognostic variable for predicting a worse outcome after surgical resection. GRP78/BiP is involved in protein folding and assembly in the ER and increased resistance to ER-stress-induced apoptosis [21,22,23], and its levels are reportedly elevated in various cancers and closely associated with malignancy, metastasis, and chemotherapy resistance [22,23]. A previous study suggested elevated GRP78/BiP level as a significant factor for predicting a favorable prognosis in patients with lung cancer [28], whereas a review indicated that elevated GRP78/BiP level correlated with drug resistance, tumor recurrence, and poor survival [23]. These findings suggest that GRP78/BiP induction could represent a therapeutic strategy targeting drug-resistant cells associated with lung, bladder, and breast cancers, whereas GRP78/BiP inhibition might be effective against resistant cells associated with gastric cancer, transformed fibroblasts, and epidermoid carcinoma. Although previous studies suggested GRP78/BiP induction or inhibition as a potential therapeutic strategy [22,23], further investigations focused on the discovery of small molecules related to modulation of ER-stress-related signaling and GRP78/BiP levels are required. Our findings indicated altered GRP78/BiP level as an ER-stress marker and involved in PPC pathogenesis and development, suggesting GRP78/BiP as a potentially promising molecular target for the treatment of PPC.

Furthermore, our data indicated that STMN1, TUBB3, TS, and Topo-II levels did not correlate significantly with OS in PPC patients; however, these markers might play an important role in PPC development. Our future work will examine relationships between currently used drugs and levels of these markers. 

This study has several limitations. First, although samples were collected from multiple institutions, the number of patients was too low to confirm our results statistically. Moreover, due to the rarity of PPC, we were unable to validate our results by using another cohort; therefore, a similar study should be performed using a larger cohort. Second, we were unable to accurately detect the *epithelial growth factor receptor* (*EGFR*)-mutation status of the primary tumors. The multi-institutional retrospective nature of this study limited the detection of the *EGFR* mutation to the physicians’ discretion; therefore, we were unable to collect complete data concerning the frequency of the mutation. Our previous study demonstrated that *EGFR* mutation was detected in ~18% of patients with PPC, especially those with an adenocarcinomatous component; however, in those with a sarcomatoid component, *KRAS* mutations were not observed in all patients [5]. Additionally, Sartori et al. reported that *EGFR* mutations were not detected in a cohort of patients with sarcomatoid carcinomas [29]. Although it remains unclear whether EGFR-tyrosine kinase inhibitors are effective for treating PPC with *EGFR* mutations, further investigation is warranted to elucidate the potential of molecular-targeting therapy. In the present study, *EGFR* mutation was not evaluated in patients with PPC; however, future studies should focus on the relationship between drug-sensitive markers and *EGFR* mutation in patients with PPC. Third, although a previous report described GRP78/BiP in human non-small cell lung cancer [30], it remains unclear whether manipulation of VEGFR2 and/or GRP78/BiP levels within PPC cells represents an efficacious strategy to decrease tumor growth. Our data indicated a close association between VEGFR2 and GRP78/BiP and PPC metastasis, tumor invasiveness, and patient survival; however, whether their targeting would increase chemotherapeutic efficacy remains unclear. Further investigations should focus on the control of VEGFR2 and GRP78/BiP levels in PPC using in vitro and in vivo studies. Although few patients in our cohort had been treated with ramucirumab, a VEGFR2 inhibitor, this might represent a promising future treatment strategy.

## 4. Materials and Methods

### 4.1. Patients

Patients with histologically confirmed PPC and who underwent surgical resection at multiple institutions between August 2001 and October 2015 (*n* = 105) were enrolled in this study. Pleomorphic carcinoma was diagnosed according to the 2015 World Health Organization Classification of Tumours [3]. Diagnoses were confirmed using light microscopy and immunohistochemistry. PPC was defined as NSCLC containing at least 10% sarcomatoid components. Surgically resected primary tumors (*n* = 105) were included in this study in accordance with institutional guidelines and the Helsinki Declaration. The institutional review boards of all participating institutions approved this study. This research has been approved by Gunma University ethic committee on 24 November 2017 (ethic code: 1385). The currently collected tumor samples used in this study were performed using those of our previous study [31].

### 4.2. Immunohistochemical Staining

Immunohistochemical staining was performed as described previously [20,32,33,34,35] and using the following antibodies: rabbit monoclonal anti-VEGFR2 (1:400; Abcam, Tokyo, Japan); mouse monoclonal anti-STMN1 (1:100; Santa Cruz Biotechnology, Dallas, TX, USA); mouse monoclonal anti-TUBB3 (1:500; MMS-435P; Convance, San Diego, CA, USA); rabbit polyclonal anti-TS (1:1600; clone RTSSA; Taiho Pharmaceutical, Saitama, Japan); rabbit polyclonal anti-Topo-II (1:100; ab180393, Abcam); anti-GRP78/BiP (1:100; Cell Signaling Technology, Danvers, MA, USA). VEGFR2 levels were assessed semi quantitatively by estimating a weighted average of the percentage of tumor cells stained on whole tumor slides (0 = no staining, 1 = positive staining in 1%–10% of the tumor cells, 2 = positive staining in 11%–25% of the tumor cells, 3 = positive staining in 26%–75% of the tumor cells, and 4 = positive staining in >75% of the tumor cells). Staining intensity was evaluated semi quantitatively by estimating the average intensity of the tumor cells (0 = no staining, 1 = weak staining, 2 = moderate staining, and 3 = strong staining and equivalent to the positive control, which showed clear, well-defined, and strong staining). The proportion and intensity scores were then added to obtain a total score, which ranged from 0 to 7 [36]. High expression was defined when tumors contained cancer cells that were assigned with a staining score of 3. STMN1 was assessed using the modified Allred score, which is a semiquantitative system that considers the proportion of positive cells (scored on a scale of 0–5: 0, none; 1, <1%; 2, 1%–10%; 3, 11%–33%; 4, 34%–66%; and 5, 67%–100%) and staining intensity (scored on a scale of 0–2: 0, none and weak; 1, intermediate; and 2, strong) [37]. The proportion and intensity were then summed to produce total scores of 0 to 7. A score of zero to two was classified as negative, whereas three through seven were positive. TUBB3 was assessed using a semiquantitative H-score. The intensity of cytoplasmic β-tubulin staining was graded on a scale of zero to two using adjacent nonmalignant cells as a reference. The percentage of positive tumor cells was evaluated, and a proportional-area score was determined (0 for 0%; 0.5 for 1%–9%; 1 for 10%–24%; 2 for 25%–49%; 3 for 50%–74%; and 4 for ≥75%). This proportion score was then multiplied by the staining intensity to obtain a final semiquantitative H-score with a range of zero to eight [33,38]. The median H-score of TUBB3 was 1.00, and the respective cutoff value was 1.00. Samples in which stained tumor cells comprised >1.00 of the H-score were graded as high-expression. TS levels were determined according the presence of nuclei or cytoplasmic staining. For TS, a semiquantitative scoring method was used according to the percentage of positive cells: 1, ≤10%; 2, 10%–24%; 3, 25%–50%; 4, 51%–75%, and 5, ≥75%. Samples in which stained tumor cells comprised >25% of the tumor were graded as high-expression [34]. Cells were deemed positive for Topo-II if positive staining was present in the nuclei. The proportion of Topo-II-positive cells was assessed using a semi-quantitative scoring method, wherein samples were assigned a score based on the percentage of positive cells: 1, <10%; 2, 10% to <25%; 3, 25% to <50%; 4, 50% to <75%; and 5, ≥75% [20]. We compared tumor response and survival data between groups showing high (Topo-II-high group) and low (Topo-II-low group) Topo-II expression, with various cut-off scores for Topo-II expression. In the present study, expression scores between one and four signified low Topo-II expression, and a score of five signified high Topo-II expression. The GRP78/BiP score was assessed by staining intensity, as follows: 1, ≤10% of the tumor area; 2, 11%–25%; 3, 26%–50%; 4, 51%–75%; and 5, ≥76%. Tumors with scores of 3, 4, or 5 were defined as exhibiting high GRP78/BiP levels [35]. Sections were evaluated using a light microscope in a blinded fashion by at least two of the authors.

### 4.3. Statistical Analysis

Statistical analyses were performed using Student’s *t*-test and the χ^2^ test for continuous and categorical variables, respectively. The Kaplan–Meier method was used to estimate survival as a function of time, and survival differences were analyzed by the log-rank test. OS represented the time from tumor resection to death from any cause, and DFS represented the time between tumor resection and the first episode of disease progression or death. Univariate and multivariate survival analyses were performed using a Cox proportional hazards model and a logistic regression model for radical surgery. A *p* < 0.05 was considered statistically significant. All statistical analyses were performed using GraphPad Prism software (v.4.0; GraphPad Software, San Diego, CA, USA) and JMP Pro software (v.12.0; SAS Institute Inc., Cary, NC, USA).

## 5. Conclusions

Analysis of resected samples from PPC patients indicated elevated levels of VEGFR2 and attenuated levels of GRP78/BiP as independent predictive factors for poor outcomes after surgical resection. These findings suggested that the management of VEGFR2 and GRP78/BiP levels might enhance the treatment of PPC.

## Figures and Tables

**Table 1:** The percentages based on differential expression of the target markers.

Markers	VEGFR2	TUBB3	STMN1	Topo-II	TS	GRP78/BiP
Average score	2.86	3.44	2.14	1.42	3.13	3.36
High-expression percentages	33% (39/105)	61% (64/105)	35% (37/105)	31% (33/105)	51% (53/105)	51% (53/105)

STMN1, stathmin 1; Topo-II, topoisomerase II; TS, thymidylate synthase; TUBB3, tubulin β3 class III; VEGFR2, vascular endothelial growth factor receptor 2.

**Table 2:** Patient demographics according to differential levels of target markers.

Variables	Total (*n* = 105)	VEGFR2			TUBB3			STMN1			Topo-II			TS			GRP78/BiP		
		High (*n* = 39)	Low (*n* = 66)	*p*	High (*n* = 64)	Low (*n* = 41)	*p*	High (*n* = 37)	Low (*n* = 68)	*p*	High (*n* = 33)	Low (*n* = 72)	*p*	High (*n* = 53)	Low (*n* = 52)	*p*	High (*n* = 53)	Low (*n* = 52)	*p*
**Age**																			
<69/≥69 (y)	54/51	25/14	29/37	0.06	34/30	20/21	0.69	21/16	33/35	0.54	23/10	31/41	0.01*	30/23	24/28	0.33	29/34	25/27	0.85
**Gender**																			
Male/Female	79/26	28/11	51/15	0.64	44/35	35/6	<0.01*	26/11	53/15	0.47	26/7	53/19	0.63	38/15	41/11	0.49	43/10	36/17	0.12
**T factor**																			
T1-2/T3-4	65/40	25/14	40/26	0.83	37/28	28/13	0.31	21/16	44/24	0.52	12/21	53/19	<0.01*	29/24	36/16	0.16	39/14	26/16	0.29
**N factor**																			
Absent/Present	72/33	27/12	45/21	>0.99	46/24	26/15	0.84	25/12	47/21	>0.99	21/12	51/21	0.50	35/18	37/15	0.67	40/13	32/1	0.01*
**Pathological stage**																			
I-II/III-IV	69/36	24/15	45/21	>0.99	41/28	28/13	0.42	20/17	49/19	0.08	25/8	44/28	0.18	34/19	35/17	0.83	41/12	28/24	0.01*
**Smoking**																			
Yes/No	84/21	28/11	56/10	0.13	51/13	33/8	>0.99	27/10	57/11	0.21	27/5	57/15	0.75	44/9	40/12	0.47	46/7	38/14	0.09
**Lymphatic permeation**																			
Absent/Present	41/64	12/27	29/37	0.21	25/16	16/25	0.07	13/24	28/40	0.67	14/19	27/45	0.67	20/33	21/31	0.84	19/34	22/30	0.55
**Vascular invasion**																			
Absent/Present	31/74	11/28	20/46	>0.99	20/11	11/30	<0.01*	10/27	21/47	0.82	10/23	21/51	>0.99	16/37	15/37	>0.99	15/38	16/36	0.83
**Pleural invasion**																			
Absent/Present	49/56	17/22	32/34	0.68	30/19	19/22	0.20	18/19	31/37	0.84	17/16	35/40	0.68	27/26	22/30	0.43	22/31	27/25	0.33
**Adjuvant chemotherapy**																			
Absent /Present	77/28	27/12	50/16	0.49	43/34	34/7	0.68	26/11	51/17	0.64	25/8	52/20	0.81	35/18	42/10	0.12	39/14	38/14	>0.99
**VEGFR2**																			
High/Low	—	—	—	—	25/39	14/27	0.52	19/18	20/48	0.03*	14/19	25/47	0.51	24/29	15/37	0.11	21/32	18/34	0.68
**TUBB3**																			
High/Low	64/41	25/14	39/27	0.52	—	—	—	17/10	37/31	0.49	18/15	46/26	0.39	41/12	23/29	<0.01*	36/17	28/24	0.16
**Stathmin 1**																			
High/Low	37/68	19/20	18/48	0.03*	27/37	10/31	0.49	—	—	—	12/21	25/47	>0.99	31/22	6/46	<0.01*	21/32	16/36	0.41
**Topo 2**																			
High/Low	33/72	14/25	19/47	0.51	18/46	15/26	0.39	12/25	21/47	>0.99	—	—	—	18/35	15/37	0.67	19/34	14/38	0.40
**TS**																			
High/Low	53/52	24/29	15/37	0.11	41/12	23/29	<0.01*	31/22	6/46	<0.01*	18/35	15/37	0.67	**-**	**-**	**-**	30/23	23/29	0.24
**GRP78/BiP**																			
High/Low	53/52	21/32	18/34	0.68	36/17	28/24	0.16	36/17	28/24	0.16	21/32	16/36	0.41	30/13	23/29	0.01*	**-**	**-**	**-**

Bold *p* values are statistically significant (*p* < 0.05). Elevated VEGFR2 level was significantly linked with STMN1 level; elevated TUBB3 level was significantly associated with gender, vascular invasion, and TS; elevated STMN1 level was significantly associated with VEGFR2 and TS levels; elevated Topo-II level was significantly associated with age and the T factor; elevated TS level was significantly associated with TUBB3, STMN1, and GRP78/BiP levels; and elevated GRP78/BiP level was significantly associated with the N factor and pathological stage. *p < 0.05 according to Student’s t-test for continuous variables and a chi-squared test for categorical variables. STMN1, stathmin 1; Topo-II, topoisomerase II; TS, thymidylate synthase; TUBB3, tubulin β3 class III; VEGFR2, vascular endothelial growth factor receptor 2.

**Table 3:** The univariate disease-free survival (DFS) and overall survival (OS) analyses.

OS												
Variables	All Patients				Patients with AC Component				Patients with Non-AC Component			
	MST	HR	95% CI	*p* Value	MST	HR	95% CI	*p* Value	MST	HR	95% CI	*p* Value
Age, ≤69/>69 (y)	361/1047	0.82	0.49–1.37	0.52	157/1038	1.34	0.59–3.04	0.48	352/1068	1.10	0.56–2.16	0.77
Gender (female/male)	1038/654	0.83	0.46–1.51	0.55	1038/1009	1.06	0.44–2.54	0.89	881/147	0.63	0.27–1.49	0.30
p-stage (I-II/III-IV)	1338/228	0.21	0.11–0.93	**<0.01**	1839/615	0.33	0.13–0.83	**0.01**	1265/145	0.07	0.02–0.18	**<0.01**
Ly (present/absent)	624/1209	1.47	0.85–2.39	0.17	1009/1939	1.22	0.52–2.83	0.64	288/1047	1.56	0.78–3.11	0.20
v (present/absent)	640/1265	1.41	0.82–2.41	0.21	1038/1839	1.16	0.49–2.72	0.72	353/1265	1.51	0.73–3.10	0.26
Pl (present/absent)	615/1202	1.56	0.93–2.61	0.09	654/3665	1.95	0.86–4.41	0.11	336/1068	1.30	0.66–2.56	0.57
VEGFR2 (high/low)	352/1209	2.23	1.28–3.83	**<0.01**	615/1839	2.23	0.98–5.02	0.05	187/1068	2.71	1.26–5.82	**0.01**
TUBB3 (high/low)	361/2010	2.15	1.27–3.62	**<0.01**	640/NR	2.07	0.91–4.68	0.08	244/1546	2.26	1.12–4.42	**0.02**
STMN1 (high/low)	359/1202	2.34	1.34–4.08	**<0.01**	291/2010	4.34	1.55–12.1	**<0.01**	507586	1.71	0.85–3.41	0.12
Topo-II (high/low)	1338/615	0.57	0.33–0.99	**0.04**	1089/1039	0.81	0.33–1.95	0.63	1338/336	0.52	0.25–1.06	0.07
TS (high/low)	361/NR	2.15	1.29–3.57	**<0.01**	624/NR	2.84	1.25–6.41	**0.01**	352/1047	1.55	0.77–3.14	0.21
GRP78/BiP (high/low)	1047/5.7	0.59	0.35–0.99	**0.04**	1657/615	0.53	0.22–1.7	0.15	991/244	0.71	0.35–1.41	0.32
**DFS**												
**Variables**	**MST**	**HR**	**95% CI**	***p***	**MST**	**HR**	**95% CI**	***p***	**MST**	**HR**	**95% CI**	***p*** **Value**
Age, ≤69/>69 (y)	267/733	0.70	0.42–1.17	0.17	266/759	1.85	0.88–1.89	0.10	247/733	1.27	0.62–2.62	0.50
Gender (female/male)	539/357	0.63	0.35–1.16	0.14	444/522	0.92	0.41–2.05	0.83	753/156	0.42	0.16–1.01	0.07
p-stage (I-II/III-IV)	890/154	0.21	0.12–0.40	**<0.01**	499/522	0.48	0.21–1.08	0.07	890/95	0.05	0.02–0.14	**<0.01**
ly (present/absent)	336/890	1.73	1.03–2.88	**0.03**	444/1235	1.45	0.68–3.09	0.32	171/836	2.00	0.97–4.13	0.06
v (present/absent)	443/1235	1.63	0.95–2.81	0.07	499/1235	1.35	0.61–2.97	0.44	267/NR	1.83	0.85–3.95	0.12
pl (present/absent)	267/765	2.04	1.22–3.39	**<0.01**	411/765	1.86	0.88–3.93	0.09	187/1068	2.71	1.26–5.82	0.03
VEGFR2 (high/low)	286/765	2.4	1.37–4.19	**<0.01**	411/654	1.71	0.81–3.61	0.16	147/912	3.98	1.75–9.05	**<0.01**
TUBB3 (high/low)	357/765	1.65	0.98–2.78	0.05	443/765	1.37	0.65–2.87	0.39	247/836	1.97	0.95–4.08	0.06
STMN1 (high/low)	336/765	2.46	1.39–4.35	**<0.01**	286/759	4.25	1.54–11.7	**<0.01**	336/NR	2.08	1.02–4.31	0.04
Topo-II (high/low)	443/522	1.03	0.59–1.78	0.92	443/539	1.38	0.61–3.13	0.60	340/336	0.90	0.42–1.91	0.78
TS (high/low)	357/753	1.57	0.94–2.61	0.08	362/759	2.14	0.99–4.64	0.05	336/753	1.22	0.58–2.56	0.59
GRP78/BiP (high/low)	444/443	1.09	0.61–1.93	0.30	1235/443	0.43	0.19–0.93	**0.03**	267/890	1.17	0.57–2.42	0.65

Bold *p* values are statistically significant (*p* < 0.05). Univariate analysis identified disease stage and STMN1 and TS levels as significant prognostic markers for OS in patients with an adenocarcinoma component, and disease stage and VEGFR2 and TUBB3 levels as significant prognostic markers for OS in patients with a non-adenocarcinoma component. Significant predictors for DFS in patients with an adenocarcinoma component were STMN1 and GRP78/BiP levels, and significant predictors for DFS in patients with a non-adenocarcinoma component were disease stage and VEGFR2 level. *p* < 0.05 is considered statistically significant according to analysis of continuous variables. AC, adenocarcinoma; CI, confidence interval; DFS, disease-free survival; HR, hazard ratio; ly, lymphatic permeation; MST, median survival time; OS, overall survival; pl, pleural invasion; STMN1, stathmin 1; Topo-II, topoisomerase II; TS, thymidylate synthase; TUBB3, tubulin β3 class III; v, vascular invasion; VEGFR2, vascular endothelial growth factor receptor 2.

**Table 4:** Multivariate OS and DFS analyses.

OS												
Variables	All Patients				Patients with AC Component				Patients with Non-AC Component			
	Univariate (*p*)	HR	95% CI	*p*	Univariate (*p*)	HR	95% CI	*p*	Univariate (*p*)	HR	95% CI	*p*
Age, ≤69/>69 (y)	0.52				0.48				0.77			
Gender (female/male)	0.55				0.89				0.30			
p-stage (I-II/III-IV)	**<0.01**	1.62	1.19–2.19	**<0.01**	**0.01**	1.67	1.03–2.81	**0.03**	**<0.01**	2.63	1.71–4.15	**<0.01**
Ly (present/absent)	0.17				0.64				0.20			
v (present/absent)	0.21				0.72				0.26			
Pl (present/absent)	0.09				0.11				0.57			
VEGFR2 (high/low)	**<0.01**	1.38	1.05–1.81	**0.01**	0.05				**0.01**	1.64	1.15–2.35	**<0.01**
TUBB3 (high/low)	**<0.01**	1.38	0.99–1.97	0.05	0.08				**0.02**	1.41	0.95–2.18	0.08
STMN1 (high/low)	**<0.01**	0.96	0.69–1.34	0.85	**<0.01**	0.92	0.51–1.66	0.78	0.12			
Topo-II (high/low)	**0.04**	1.13	0.83–1.59	0.43	0.63				0.07			
TS (high/low)	**<0.01**	1.43	0.98–2.11	0.05	**0.01**	1.71	0.99–2.99	0.05	0.21			
GRP78/BiP (high/low)	**0.04**	1.36	1.03–1.81	**0.02**	0.15				0.32			
**DFS**												
**Variables**	**Univariate** (***p***)	**HR**	**95% CI**	***p***	**Univariate** (***p***)	**HR**	**95% CI**	***p***	**Univariate** (***p***)	**HR**	**95% CI**	***p***
Age, ≤69/>69 (y)	0.17				0.10				0.50			
Gender (female/male)	0.14				0.83				0.07			
p-stage (I-II/III-IV)	**<0.01**	1.67	1.26–2.23	**<0.01**	0.07				**<0.01**	3.07	1.96–5.29	**<0.01**
ly (present/absent)	**0.03**	0.95	0.71–1.28	0.77	0.32				0.06			
v (present/absent)	0.07				0.44				0.12			
pl (present/absent)	**<0.01**	1.33	1.02–1.77	**0.03**	0.09				0.03			
VEGFR2 (high/low)	**<0.01**	1.36	1.05–1.76	**0.01**	0.16				**<0.01**	1.83	1.26–2.66	**<0.01**
TUBB3 (high/low)	0.05				0.39				0.06			
STMN1 (high/low)	**<0.01**	1.33	1.01–1.74	**0.03**	**<0.01**	1.94	1.26–2.94	**<0.01**	0.04			
Topo-II (high/low)	0.92				0.60				0.78			
TS (high/low)	0.08				0.05				0.59			
GRP78/BiP (high/low)	0.30				**0.03**	1.66	1.13–2.46	**0.01**	0.65			

Bold *p* values are statistically significant (*p* < 0.05). Multivariate analysis confirmed the disease stage as an independent prognostic factor for predicting worse OS in patients with an adenocarcinoma component and disease stage and VEGFR2 level as independent prognostic factors for predicting worse OS in patients with a non-adenocarcinoma component. Significant prognostic markers for DFS in patients with an adenocarcinoma component were STMN1 and GRP78/BiP levels and disease stage and VEGFR2 level in patients with a non-adenocarcinoma component. *p* < 0.05 is considered statistically significant according to analysis of continuous variables. AC, adenocarcinoma; CI, confidence interval; DFS, disease-free survival; HR, hazard ratio; ly, lymphatic permeation; MST, median survival time; OS, overall survival; pl, pleural invasion; STMN1, stathmin 1; Topo-II, topoisomerase II; TS, thymidylate synthase; TUBB3, tubulin β3 class III; v, vascular invasion; VEGFR2, vascular endothelial growth factor receptor 2.
